# Case Report: Primary choriocarcinoma of the pineal region

**DOI:** 10.3389/fonc.2026.1822123

**Published:** 2026-05-29

**Authors:** Mengxing Wu, Wei Xia

**Affiliations:** 1 Wuhan Children's Hospital (Wuhan Maternal and Child Healthcare Hospital), Wuhan Maternal and Child Healthcare Hospital, Wuhan University of Science and Technology, Postgraduate Training Base, Wuhan, China; 2 School of Medicine, Wuhan University of Science and Technology, Wuhan, China; 3 Wuhan Children's Hospital (Wuhan Maternal and Child Healthcare Hospital), Tongji Medical College, Huazhong University of Science and Technology, Wuhan, China

**Keywords:** children, magnetic resonance imaging, pineal region tumor, primary intracranial choriocarcinoma, β-human chorionic gonadotropin

## Abstract

Primary intracranial choriocarcinoma (PICCC) is a rare, highly malignant non-germinomatous germ cell tumor (NGGCT), primarily affecting children and adolescents with a male predominance. It most commonly arises in the pineal region and sella turcica. This report describes a case of primary choriocarcinoma in the pineal region. CT imaging revealed a patchy area of abnormal high density with slight internal density variation and small nodular calcifications. MRI showed a mixed signal pattern, predominantly slightly short T_1_-weighted and short T_2_-weighted signals. Contrast-enhanced imaging demonstrated heterogeneous enhancement. Surgical pathology specimens showed immunohistochemical positivity for HCG(+), HPL(+), CK(PAN)(+), EMA(partial+), p53(partial+), PLAP(placental alkaline phosphatase, partial+), CD117(partial+), and Ki-67 proliferation index: 20%. The pathological findings were consistent with pineal region choriocarcinoma. The patient’s serum β-HCG level decreased to <0.1 mIU/mL one year after surgery, and the child remained in good condition without recurrence during the 2-year follow-up.

## 1 Introduction

PICCC is a rare, highly malignant NGGCT that accounts for approximately 3%–5% of intracranial germ cell tumors and predominantly affects children and adolescents, with a male predominance (1). Only a limited number of studies have described its imaging features, which exhibit heterogeneity due to the presence of pathological components such as hemorrhage and necrosis (2), making differentiation from other pineal region tumors challenging. Compared to other types of germ cell tumors (3), choriocarcinoma demonstrates poorer survival rates, underscoring the critical importance of early diagnosis. This paper presents a retrospective analysis and discussion of clinical data and imaging features in a pediatric patient with pathologically confirmed primary pineal region choriocarcinoma, aiming to enhance clinical awareness of this disease.

## 2 Case presentation

A previously healthy 9-year-6-month-old male child was admitted with complaints of dizziness, headache, blurred vision, and vomiting for more than 22 hours. Physical examination revealed no abnormalities. Head CT at another hospital showed abnormal density in the pineal region, suggestive of a germ cell tumor. Laboratory tests at our hospital revealed significantly elevated serum β-human chorionic gonadotropin (β-HCG) at 1017.00 mIU/mL (normal reference range <5 mIU/mL); serum alpha-fetoprotein (AFP) was negative.

Non-contrast head CT performed at our institution (**Figure 1**) demonstrated a patchy hyperdense lesion in the pineal region with slightly heterogeneous density and small nodular calcifications. Bilateral lateral ventricles and the third ventricle were mildly enlarged, with small hyperdense foci noted, consistent with a pineal region tumor complicated by intraventricular hemorrhage.

**Figure 1:**
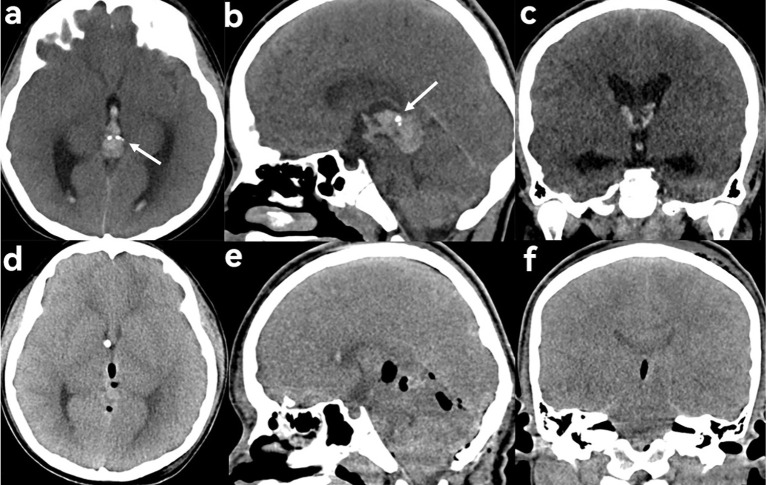
Choriocarcinoma of the pineal body in a 9-year-old male patient presenting with dizziness and headache. **(a–c)** Preoperative CT showed a patchy hyperdense, slightly inhomogeneous lesion in the pineal body with small nodular calcification (white arrow); CT values range from 30 to 119 Hu, and the lesion measures approximately 30 mm × 20 mm × 16 mm. Mild enlargement of the bilateral lateral and third ventricles was noted, along with intraventricular hyperdensity (66 Hu) suggestive of hemorrhage. **(d–f)** Postoperative CT scans obtained one week later showed complete resection of the lesion, with postoperative changes in the pineal region.

Brain MRI with and without contrast (**Figure 2**) showed a mixed-signal mass in the pineal region, predominantly exhibiting slightly shortened T_1_ and T_2_ signals. The lesion had relatively well-defined borders, measuring approximately 15 mm × 37 mm × 19 mm, with involvement of the bilateral dorsal thalamus and midbrain tectum, resulting in compression and narrowing of the cerebral aqueduct. Bilateral lateral ventricles and the third ventricle were mildly enlarged. Patchy areas of long T_1_ and long T_2_ signal edema were visible in the periventricular white matter, and patchy slightly shortened T_2_ signals were noted along the ventricular walls. Post-contrast imaging revealed heterogeneous enhancement of the mass, with no significant diffusion restriction observed on DWI (**Figure 3**).

**Figure 2:**
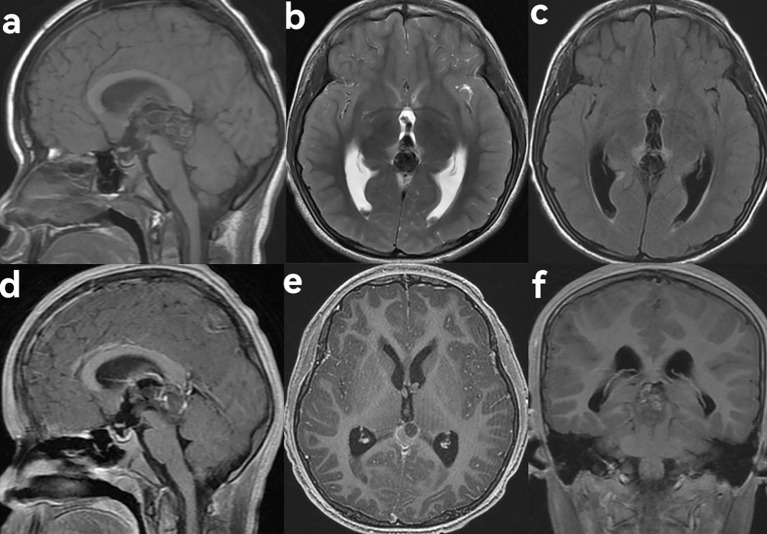
MRI findings of pineal region choriocarcinoma in a 9-year-old male patient presenting with dizziness and headache. **(a)** Sagittal T_1_WI showed a slightly hyperintense mass in the pineal region, with compression of the cerebral aqueduct and involvement of the bilateral dorsal thalami and midbrain. **(b)** Axial T_2_WI showed hypointense signal with patchy short T_2_ foci within the ventricles. **(c)** T_2_-FLAIR showed periventricular edema. **(d–f)** Contrast-enhanced sagittal, axial, and coronal images showed heterogeneous enhancement of the mass.

**Figure 3:**
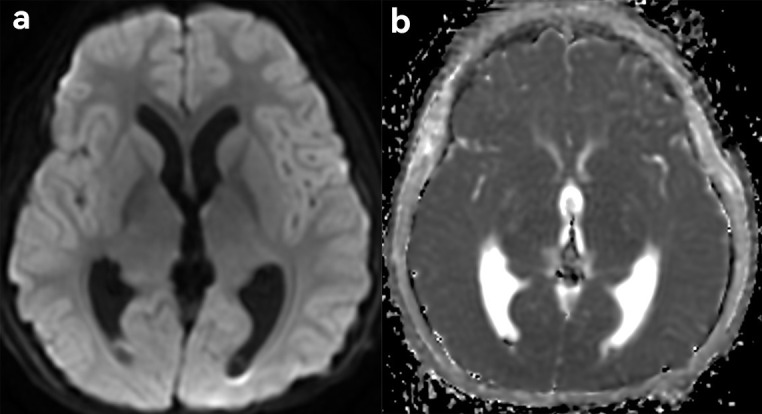
Diffusion-weighted imaging features (DWI). **(a)** On DWI, the solid component of the lesion appeared hypointense. **(b)** On apparent diffusion coefficient (ADC) mapping, the solid component appeared heterogeneously slightly hyperintense. No significant diffusion restriction was observed in the mass.

The patient subsequently underwent pineal tumor resection and ventriculostomy for external ventricular drainage, which was performed to relieve preoperative obstructive hydrocephalus. Postoperative pathology confirmed the diagnosis of pineal region choriocarcinoma (**Figure 4**). Immunohistochemical staining showed: HCG(+), HPL(+), CK(PAN)(+), PLAP(partial+), Ki-67 proliferation index of 20%, H3K27me3(+), INI-1(+), and AFP(-).

**Figure 4:**
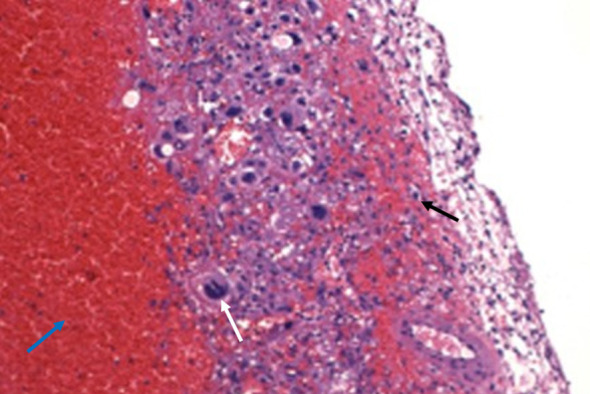
Pathological findings. Hemorrhage (blue arrow) and necrosis were observed within the lesion, with visible syncytiotrophoblasts (white arrow) and cytotrophoblasts (black arrow) showing marked cellular atypia.

Postoperatively, the patient developed restricted upward gaze and impaired convergence reflex. One week after surgery, serum β-HCG decreased to 16.35 mIU/mL. According to the 2018 Chinese Expert Consensus on the Diagnosis and Treatment of Primary Central Nervous System Germ Cell Tumors, the patient received adjuvant chemotherapy tailored for non-germinomatous germ cell tumors. Alternating IE (Ifosfamide + Etoposide) and EP (Etoposide + Cisplatin) regimens were administered from October 2021 to February 2022. The patient then received radiotherapy from March 1 to April 6, 2022, delivering a total dose of 48.6 Gy in 28 fractions, followed by continuation of the chemotherapy regimen and regular follow-up. Serum β-HCG dropped to <0.1 mIU/mL one year postoperatively. At the 2-year follow-up, the patient recovered well, with no evidence of recurrence on serial spinal and cranial MRI examinations performed at our hospital and external institutions.

## 3 Discussion

### 3.1 Overview

Central nervous system germ cell tumors (GCTs) represent a rare category of primary intracranial tumors, accounting for 3%–11% of pediatric intracranial tumors and only approximately 1% of adult intracranial tumors. Based on histological characteristics, GCTs are classified into two main categories: germinomatous germ cell tumors and NGGCTs. NGGCTs account for approximately one-third of all intracranial GCTs and include subtypes such as teratoma, embryonal carcinoma, yolk sac tumor, choriocarcinoma, and mixed germ cell tumor (4–6).

Among these, PICCC represents one subtype of NGGCTs, accounting for approximately 3%–5% of primary intracranial GCTs (7). It is one of the rarest and most malignant subtypes of primary intracranial GCTs, characterized by ectopic differentiation along the trophoblastic cell line and significantly elevated serum and cerebrospinal fluid β-HCG levels (2). It predominantly affects children and adolescents, with a male predominance, and most commonly arises in the pineal and sellar regions (1). Derived from primordial germ cells, PICCC is characterized by aggressive invasiveness, a high tendency for hemorrhage, early cerebrospinal fluid dissemination, and markedly elevated β-HCG levels in both serum and cerebrospinal fluid (2, 8). Most patients present with non-specific symptoms such as headache, vomiting, and visual disturbances, which may be accompanied by endocrine abnormalities (2, 3, 7). The pediatric patient in this case presented with dizziness, headache, blurred vision, and vomiting as the main clinical manifestations. Notably, despite significantly elevated serum β-HCG levels, the patient did not exhibit signs of precocious puberty, which may be attributed to the patient’s young age, insufficient biological activity of the tumor-secreted β-HCG, and early surgical intervention that limited the duration of hormonal exposure before clinical manifestations developed.

### 3.2 Imaging findings

When PICCC is suspected, imaging features play a critical role in preoperative diagnosis. Literature reports describe pineal-region PICCC as typically appearing on plain CT as a solitary, round, high-density soft tissue mass (often due to hemorrhage) with heterogeneous density, clear margins, and frequent punctate or granular calcifications. However, as most reports are case-based, these findings remain nonspecific. The present case showed small nodular calcifications in the pineal region, a feature whose diagnostic value remains controversial (2, 7).

On MRI, PICCC commonly presents as a heterogeneous mixed-signal lesion reflecting its diverse components, including hemorrhage, fibrosis, necrosis, and vascular proliferation (2, 5, 9). Consistent with the literature, our patient’s lesion demonstrated predominantly short T_2_-weighted mixed signals, with low signal areas suggesting hemosiderin deposition from chronic hemorrhage or calcification (2). On T_1_WI, the lesion showed mixed low signal intensity, with patchy short T_1_ high signal foci corresponding to subacute hemorrhage, a hallmark feature of this trophoblastic tumor. Necrotic and cystic areas, which appear as long T_1_/long T_2_ signals, were also observed (7).

PICCC exhibits malignant invasiveness: although tumor margins may appear well-defined, the lesion frequently invades adjacent structures, most commonly the thalamus and midbrain (2). In our case, bilateral dorsal thalami and midbrain tectum were involved. Dissemination via cerebrospinal fluid is also common, manifesting as enhancing nodules along ventricular walls and in the subarachnoid space. Abnormal nodular slightly short T_2_ signals in the ventricular system strongly suggested such dissemination (**Figure 3**).

On contrast-enhanced scans, most cases show marked heterogeneous enhancement of solid components, with necrotic areas remaining unenhanced; a “garland-like” enhancement pattern has also been described in a minority of cases. In our patient, DWI showed low signal intensity in the solid components, with mixed to slightly increased ADC values and no significant diffusion restriction, likely due to obscuration by hemorrhagic foci. The mass itself demonstrated heterogeneous enhancement. Secondary changes, including obstructive hydrocephalus from midbrain aqueduct compression, periventricular edema, and protein deposition, were also present, consistent with the mass effect of the lesion (7, 10).

### 3.3 Differential diagnosis

Choriocarcinoma and germ cell tumors are both associated with elevated β-HCG expression (11). Furthermore, before considering a diagnosis of PICCC, hemorrhagic metastases from the reproductive system must be ruled out. This is because extracranial choriocarcinoma, particularly that of gonadal origin, is known to spread via the vascular route and frequently metastasizes to the central nervous system in 3%–28% of patients (2).

PICCC in the pineal region requires differential diagnosis from the following tumors: (1) Pineal region germ cell tumor: CT often shows encasement of pineal physiological calcifications; MRI demonstrates isointense T_1_, isointense/hypointense T_2_ signal with uniform and marked enhancement on contrast-enhanced imaging; hemorrhage is rare. Biomarkers: Serum/CSF AFP mildly elevated or normal; β-HCG typically shows no significant elevation. (2) Embryonal carcinoma: Although exhibiting mixed signal intensity, it shows marked perilesional edema. Cystic necrosis is common, but hemorrhage occurs less frequently than in PICCC. Biomarkers: AFP is markedly elevated, β-HCG may be mildly elevated. (3) Metastatic choriocarcinoma: β-HCG also markedly elevated, but with history of primary choriocarcinoma in uterus/testis; PICCC is intracranial primary with no primary reproductive tract lesion. Imaging features are similar and may be multiple (PICCC typically solitary), requiring differentiation based on medical history. (4) Pineal Region Glioblastoma: Extremely marked perilesional edema; large areas of high signal on T_2_WI/FLAIR; marked diffusion restriction on DWI (unlike PICCC without significant diffusion restriction); hemorrhage is rare and often associated with necrosis. Biomarkers: β-HCG and AFP are normal.

PICCC diagnosis relies on histopathology and immunohistochemistry. Characteristic histology shows coexistence of biphasic trophoblastic cell components—including cytotrophoblast cells and syncytiotrophoblastic giant cells. This morphology fundamentally differs from other pineal tumors, such as the nests of small cells in germ cell tumors or the primitive neuroepithelial cells in pineoblastomas, serving as the core basis for pathological diagnosis. Immunohistochemistry: Syncytiotrophoblast giant cells express human chorionic gonadotropin (HCG). Of note, this patient showed focal (partial) positivity for PLAP, a specific marker for germinomas, indicating the presence of minor germinoma components within the tumor. This finding is consistent with the characteristic of PICCC, which frequently coexists with other germ cell tumor components (e.g., germinoma, teratoma) to form mixed germ cell tumors. Furthermore, the Ki-67 proliferation index in this case was 20%, which is lower than the commonly reported level (>50%) for PICCC in previous literature. This discrepancy may be attributed to the histopathological sampling site, consistent with the variable Ki-67 expression in PICCC due to tumor heterogeneity (8). This does not compromise the accuracy of the pathological diagnosis, as the core diagnostic basis remains the biphasic trophoblastic cell morphology and the positive expression of specific markers such as HCG and HPL.

### 3.4 Treatment and prognosis

Pineal region choriocarcinoma originates from primitive germ cells, predominantly as primary tumors with a minority being metastatic (secondary to gynecological or testicular choriocarcinoma). Its biological characteristics include high invasiveness, susceptibility to hemorrhage, and potential early dissemination via cerebrospinal fluid, resulting in a poor prognosis (3, 8). Choriocarcinoma secretes β-HCG, often resulting in markedly elevated serum β-HCG levels. Studies indicate patients with significantly elevated serum β-HCG (>5000 mIU/mL) are highly likely to have choriocarcinoma (2). This disease exhibits high drug resistance and low sensitivity to radiotherapy alone, with traditional treatments yielding suboptimal outcomes. In the absence of biopsy, typical radiological features and elevated β-HCG provide strong diagnostic guidance (1). The preferred clinical approach is comprehensive treatment combining total tumor resection with chemoradiotherapy, which often yields favorable outcomes (2, 3, 12). Surgical goals include relieving hydrocephalus, confirming pathological diagnosis, and achieving maximal tumor resection. Following surgery and other standardized treatments, β-HCG levels typically return to normal ranges. Jiang et al. (8) demonstrated that serum β-HCG and alpha-fetoprotein (AFP) serve as validated biological markers in PICCC patients—normalization of β-HCG post-treatment (<0.1 mIU/mL) indicates effective tumor clearance, while its recurrence (or persistent elevation) strongly suggests tumor relapse or cerebrospinal fluid (CSF) dissemination. For mixed germ cell tumors with choriocarcinoma components, AFP serves as a supplementary marker. Combined monitoring of both markers enables more comprehensive assessment of treatment efficacy and early detection of recurrence (12). This patient remained recurrence-free at 2-year follow-up after comprehensive treatment, with serum β-HCG returning to normal levels. This case suggests that early diagnosis and standardized therapy may contribute to a favorable clinical outcome. The postoperative ocular motor disorder (due to mesencephalic tectum injury) resolved with rehabilitation therapy, underscoring the need for meticulous protection of critical surrounding neural structures during surgery to minimize complications.

### 3.5 Limitations

This case report has several limitations that should be acknowledged. First, as a single-center and single-case study, the findings have limited generalizability, and it is impossible to establish a definite causal relationship between the intervention strategy and long-term prognosis. Second, this case presented atypical pathological manifestations compared with previously reported PICCC. The Ki-67 proliferation index was only 20%, obviously lower than the commonly reported cutoff of over 50%. Such inconsistency may be attributed to tumor heterogeneity and pathological sampling bias.

## 4 Conclusion

In summary, PICCC exhibits characteristic imaging features, but its extremely rare occurrence and high malignancy result in poor prognosis. Early diagnosis is critical for patient prognosis. When presenting adolescents exhibit signs of precocious puberty alongside markedly elevated serum β-HCG levels, combined with imaging findings indicating a lesion in the sella turcica or pineal region, intra-tumoral hemorrhage (possibly with calcifications), and heterogeneous, marked enhancement on contrast-enhanced imaging, high suspicion for PICCC is warranted (7). Due to the tumor’s rich vascularity, stereotactic biopsy carries a 20–30% bleeding risk and poses a high risk of intratumoral hemorrhage and neurological/cerebrospinal fluid metastasis; thus, preoperative biopsy is not recommended (2). However, for patients with pineal region tumors complicated by obstructive hydrocephalus, neuroendoscopic biopsy combined with endoscopic third ventriculostomy (ETV) is increasingly considered a safe and effective alternative to obtain a histological diagnosis while relieving hydrocephalus (13).

## Data Availability

The original contributions presented in the study are included in the article/supplementary material. Further inquiries can be directed to the corresponding author.
